# Concentrations of Retinol and α-Tocopherol in Tissue Samples From Anna's Hummingbirds (*Calypte anna*)

**DOI:** 10.3389/fvets.2020.00637

**Published:** 2020-09-29

**Authors:** Stephanie M. Diao, Robert H. Poppenga, Gwendolyne Gonzales Alarcio, Janet E. Foley, Ruta R. Bandivadekar, Linda S. Aston, Lisa A. Tell

**Affiliations:** ^1^Department of Medicine and Epidemiology, School of Veterinary Medicine, University of California, Davis, Davis, CA, United States; ^2^California Animal Health and Food Safety Laboratory System, University of California, Davis, Davis, CA, United States

**Keywords:** retinol, α-tocopherol, hummingbird, analytical method, vitamin concentrations, tissue samples

## Abstract

Retinol (vitamin A) and α-tocopherol (vitamin E) concentrations were measured in tissue samples (liver, heart, pectoral muscle, and brain) from Anna's Hummingbirds (*Calypte anna*). Hummingbirds were after-hatch year birds that were sourced from various rehabilitation centers throughout California. Tissues samples were analyzed using high-performance liquid chromatography (HPLC). Minimum, maximum, mean, standard deviation (SD), and median ppm concentrations were calculated for each vitamin and tissue sample type. A novel analytical method was developed to analyze small mass tissue samples, with the smallest sample mass being 0.05 g for which analysis can be performed. Mean ± standard deviation (SD) concentrations of retinol in hummingbird livers, hearts, and pectoral muscle samples were 269.0 ± 216.9 ppm, 1.8 ± 2.2 ppm, and 0.3 ± 0.1 ppm, respectively. Mean ± SD α-tocopherol concentrations were 6.9 ± 4.6 ppm, 5.5 ± 4.0 ppm, 3.7 ± 2.2 ppm, and 9.1 ± 3.2 ppm for liver, heart, pectoral muscle, and brain samples, respectively. Vitamin concentrations from varying tissue types were compared to determine which were best associated with liver concentrations, the most commonly analyzed tissue for these vitamins. For both retinol and α-tocopherol, heart samples were most strongly associated with the liver samples. The results of this study provide baseline retinol and α-tocopherol concentrations in different tissue types from Anna's hummingbirds. These baseline values may be utilized in conservation efforts to avoid hypervitaminosis and hypovitaminosis of rehabilitated and/or captive hummingbirds by providing guidelines for nutritional targets which could be assessed on post-mortem examinations. Post-mortem examination of birds and measurement of vitamin concentrations in tissues may allow for dietary changes that aid captive hummingbirds.

## Introduction

As avian pollinators, hummingbirds play an important role in maintaining the health of a diverse ecosystem (1). Unfortunately, at least 26 hummingbird species are threatened (2). With a decrease in pollinators, there is an increased risk of crop failure and loss of biodiversity (2). Thus, successful rehabilitation of injured or sick hummingbirds and maintenance of healthy captive populations have the potential to be important from a holistic perspective.

Nutritional disorders are the most common non-infectious type of disease described in captive hummingbirds (3). Since hummingbirds have a unique diet of insects and nectar, replicating a hummingbird's diet can be quite difficult. This becomes an issue when hummingbirds enter rehabilitation centers and are fed a prepared commercial diet for other avian species. Many prepared diets are formulated based on the nutritional requirements of poultry (4) which can lead to hypervitaminosis or hypovitaminosis when used for other avian species. Establishing baseline vitamin concentrations in hummingbird tissues may help with formulating more appropriate diets or assess the adequacy of a given diet. However, little research has been conducted on hummingbirds' nutrient requirements (5).

Retinol (vitamin A) and α-tocopherol (vitamin E) in particular may be essential for hummingbird health during the rehabilitation process. Retinol is needed for normal metabolism, cell differentiation, and growth; α-tocopherol is an antioxidant that helps remove free radicals produced from lipid oxidation, metabolism, and tissues under stressful conditions (6, 7).

Analytical methods for measuring tissue vitamin concentrations exist for a variety of species, although most current methods require at least 0.25 g of tissue for α-tocopherol analysis (8) and 0.5 g of liver for retinol analysis^1^. Given that the average Anna's Hummingbird (*Calypte anna*) total body mass is only 4–4.5 g^2^, obtaining an adequate amount of tissue for vitamin analysis would be challenging, especially for liver and heart samples which weigh on average 0.07 and 0.09 g, respectively (Supplemental Table 1).

The objective of this study was to establish baseline retinol and α-tocopherol tissue concentrations in the Anna's Hummingbird, *C. anna*, and compare vitamin concentrations in different tissues with liver sample concentrations. To achieve this goal, a high-performance liquid chromatography (HPLC) analytical method able to quantify retinol and α-tocopherol in small mass tissue samples (as little as 0.05 g) was developed and validated. This new analytical method was used to quantify retinol and α-tocopherol concentrations in liver, heart, pectoral muscle, and brain samples from deceased hummingbirds.

## Materials and Methods

### Tissue Samples

Carcasses of 40 Anna's Hummingbirds were obtained from the following wildlife rehabilitation centers: Lindsay Wildlife Center, California Wildlife Center, Santa Barbara Wildlife Care Network, and Wetlands and Wildlife Care Center of Orange County. Only after-hatch year (AHY) birds were utilized in this study. Sex, species, and age were determined as described by The North American Banders' Manual for Hummingbirds (9). Whole brain, liver, heart, and pectoral muscle samples were collected from the carcasses using Iris scissors. Tissues were immediately placed in individual 1.5 mL cryogenic tubes (Thermo Fisher Scientific, Waltham, WA) and stored at −20°C. Tissue (liver, heart, pectoral muscle) samples from 20 hummingbirds (10 females and 10 males) were used for the retinol analysis and tissue (liver, heart, pectoral muscle, and brain) samples from 20 other hummingbirds (10 females and 10 males) were used for α-tocopherol analysis at the California Animal Health and Food Systems Laboratory (CAHFS) in Davis, California. Care was taken to ensure that samples were exposed to the least amount of light possible. Permission for obtaining the hummingbird carcasses for scientific study was approved by the United States Fish and Wildlife Service (Permit: MB55944B-2) and the California Department of Fish and Wildlife (Permit: SC-013066).

### Chemicals and Reagents

Retinol (all-trans-retinol, >97% purity) and α-tocopherol (DL-alpha-tocopherol, >97% purity), butylated hydroxytoluene (BHT), and ascorbic acid were purchased from Sigma-Aldrich (St. Louis, MO). BHT and ascorbic acid were used as antioxidants for vitamins A and E, respectively. Ascorbic acid (1%) in ethanol was prepared by dissolving 1.0 g of ascorbic acid in 100 mL of ethanol. BHT (1%) in alcohol was prepared by dissolving 1.0 g of BHT in 100 mL of methanol (used for long term storage of retinol stock standard solutions) or ethanol (used during the α-tocopherol extraction process). HPLC grade methanol, pesticide grade petroleum ether, and potassium hydroxide (KOH) pellets were purchased from Thermo Fisher Scientific (Waltham, WA). Saturated KOH was prepared by adding KOH pellets to a volume of water until precipitation persists. Optima grade, Koptec Pure Ethanol-200-proof was purchased from Decon Laboratories, Inc. (King of Prussia, PA). 18 MΩ water (Aqua Solutions Water Purification System (Jasper, GA) was used throughout the study.

### Standard Preparations

The retinol stock standard solution of 1,000 μg/mL was prepared by weighing 25 mg into a 25 mL amber volumetric flask and bringing up to volume with 1% BHT in methanol. All intermediate standards of retinol were made by serial dilutions in 1% BHT in methanol. The α-tocopherol stock standard 1,000 μg/mL was made by weighing 50 mg neat material into a 50 mL volumetric flask and bringing up to volume with methanol. All intermediate standards were made by serial dilutions in methanol. Stock standard solutions and intermediate solutions for both vitamins were stored at −20°C in amber vials to protect from light-induced oxidation. Calibration curves were prepared fresh daily, in methanol, from the intermediate standards.

### Chromatographic Conditions

An Agilent 1200 high-performance liquid chromatography (HPLC) system equipped with 1260 Infinity FLD fluorescence detector (Agilent, Santa Clara, CA) and ChemStation software to control the system were used for the quantitative analysis of both vitamins. For retinol analysis, a Spherisorb ODS2 4.6 × 250 mm, 5 μm particle size (Waters Corporation, Milford, MA) reverse phase column was used. Separation was achieved with isocratic elution using mobile phases consisting of water (solvent A) and methanol (solvent B) at 4:96 ratio. The flow rate was 1 ml/min and total run time was 10 min. Detection was performed using a fluorescence detector with excitation and emission wavelengths at 300 and 470 nm, respectively.

For α-tocopherol analysis, a Spherisorb ODS2 4.6 × 150 mm, 5 μm particle size (Waters Corporation, Milford, MA) reverse phase column was used. A single mobile phase consisting of 100% methanol was used (isocratic) at a flow rate of 1 mL/min. The total run time was 9 min. The fluorescence detector was set to excitation and emission wavelengths of 295 and 340 nm, respectively. The column temperature for both analyses was ambient.

### Sample Preparation for Retinol Analysis

Vitamin A was analyzed as total retinol. For liver and heart tissue, sample preparation began by weighing each tissue (wet mass, Supplemental Table 1) into individual 50 mL disposable, plastic test tubes. For pectoral muscle tissue, 0.1 g (wet mass) was weighed out into individual 50 mL disposable, plastic test tubes. Ascorbic acid (1%) in ethanol was added to each tube until the total mass was 2.5 g. The sample was vortexed briefly and then homogenized using a GenoGrinder (SPEX Sample Prep, Metuchen, NJ) set to 750 rpm for 5 min with 2 steel grinding balls. A 1.25 g aliquot of the homogenate was weighed into a glass, disposable, screw cap test tube. One milliliter of water, 0.75 mL of 1% ascorbic acid in ethanol, and 150 μL of saturated KOH were added, and the samples were saponified in a 70°C water bath for 20 min. After the samples were cooled to room temperature, 10 mL of petroleum ether was added to each tube. The samples were then vortexed for 1 min followed by centrifugation at 1,300 × g for 3 min. A 5 mL aliquot of each supernatant was transferred to a clean test tube and evaporated to dryness by a gentle flow of nitrogen at 35–45°C. The residue was reconstituted in 0.2 mL of methanol. The extract was sonicated, vortexed, filtered through a 0.45 μm Millipore syringe filter into an autosampler vial, and submitted for HPLC analysis.

### Sample Preparation for α-Tocopherol Analysis

Vitamin E was analyzed as α-tocopherol. For liver and heart tissues, sample preparation began by weighing each tissue (wet mass) into individual 50 mL disposable, plastic test tubes (Supplemental Table 8). 0.5 mL of 1% BHT in ethanol was added to the sample and vortexed. After the addition of 5 mL of petroleum ether, each sample was homogenized using a GenoGrinder and 2 steel grinding balls at 750 rpm for 5 min. The samples were then centrifuged at 1,300 × g for 3 min and the supernatant collected and filtered through Whatman 1PS filter paper. A 2.5 mL aliquot was transferred into a clean test tube and evaporated to dryness under a gentle flow of nitrogen at 35–45°C. The extract was then reconstituted in 0.2 mL of methanol, which was then sonicated, vortexed, filtered through a 0.45 μm Millipore syringe filter into an autosampler vial, and submitted for HPLC analysis.

The same procedure was followed for brain and pectoral muscle with the following modifications: 0.2 ml of 1% BHT in ethanol was added to a whole brain tissue. One milliliter of 1% BHT in ethanol was added to 0.1 g (wet mass) of pectoral muscle and extracted with 10 mL of petroleum ether. A 5 mL aliquot was taken, and the evaporated sample was reconstituted in 1 mL of methanol. Once placed into autosampler vials, extracts of each of the four tissue types were treated in an identical manner.

### Quality Control Sample Preparation and Method Validation

Method validation was performed using chicken tissue homogenates. Quality control sample collection and preparation began by removing tissue (liver, heart, pectoral muscle, and brain) samples from 3 chickens (*Gallus gallus domesticus*) procured from the Hopkins Avian Facility at University of California, Davis. Each tissue type was composited and homogenized using a blender. Two gram aliquots of tissue homogenate were stored in foiled packets at −20°C until analysis.

Multiple sub-aliquots of 0.1 and 0.05 g (wet mass) of liver, heart, and pectoral muscle homogenates were analyzed for both vitamins. 0.05 g aliquots of brain homogenates were analyzed for α-tocopherol only, using the same procedures described above. Established ranges of both vitamins in each of the tissues collected were determined from results of the method validation. Established ranges, defined as the mean concentration ± 2 standard deviations, were used for quality control (QC) purposes.

The established ranges measured for retinol were 482.2 ± 115.3 ppm for liver (*n* = 11), 0.2 ± 0.1 ppm for heart (*n* = 15), and 0.3 ± 0.06 ppm for pectoral muscle (*n* = 14) (Supplemental Table 2). For α-tocopherol, the established ranges were 9.3 ± 1.3 ppm for liver (*n* = 11), 8.3 ± 2.7 ppm for heart (*n* = 11), 1.2 ± 0.1 ppm for pectoral muscle (*n* = 11), and 6.2 ± 0.9 ppm for brain (*n* = 9) (Supplemental Table 2). Intra-assay and inter-assay variability (% CV) are reported in Supplemental Tables 3, 4 for retinol and α-tocopherol, respectively.

Analyte recoveries were determined by spiking chicken tissue homogenates with a known amount of each vitamin and processing the samples as outlined above. Aliquots of homogenates (*n* = 8 of each vitamin for each tissue, *n* = 6 of α-tocopherol for brain) were fortified at 500 ppm retinol and 10 ppm α-tocopherol for liver, 1 ppm retinol and 10 ppm α-tocopherol for heart, 1 ppm retinol and 10 ppm α-tocopherol for pectoral muscle, and 10 ppm α-tocopherol for brain. After analysis, the peak area for each vitamin in the unspiked sample was subtracted from the peak area of the spiked sample. Recovery was calculated as the percentage of the corrected concentrations of the spiked amount. The spike recoveries, as well as the mean, standard deviation, % CV are listed in Supplemental Tables 5, 6.

To measure absolute recovery of each vitamin, solvent spikes were made by weighing 0.1 g of the 1 and 500 μg/ml retinol standards and 0.1 g of the 1 μg/ml α-tocopherol standard into separate empty tubes and processed using the same procedures described above. The mean recoveries, standard deviations, and ranges for the solvent spikes (*n* = 5) for each matrix are reported in Supplemental Table 2. The established concentration range for the retinol 1 ppm and 500 ppm solvent spikes were 1.1 ± 0.2 ppm and 513.3 ± 106.7 ppm, respectively. The established range for α-tocopherol at 1 ppm was 1.0 ± 0.2 ppm. Intra-assay and inter-assay variability for both retinol and α-tocopherol solvent spikes are reported in Supplemental Tables 3, 4.

Each analytical batch of hummingbird samples was extracted and analyzed with QC samples consisting of duplicate chicken tissue homogenates and a solvent spike at 1 and/or 500 ppm. Based on the results from the analyses of chicken tissue homogenates, the concentration of α-tocopherol, in all of the hummingbird tissue samples, and retinol, in hummingbird pectoral muscle samples, were expected to be low (<10 ppm) that only a 1 ppm solvent spike was used. For retinol analyses of heart and liver hummingbird samples, both 1 and 500 ppm solvent spikes were used as QC.

Standard quality assurance requirements for any batch involved an *R*^2^ of the standard curve equal to or >0.980 and an analyte recovery of the solvent spikes and chicken tissue homogenates to be within the previously established ranges for both vitamins.

### Data Analysis

Vitamin quantitation was accomplished by using a 10- to 16-point curve at concentrations of 0.25, 0.5, 1.0, 10, 20, 30, 40, and 50 ng of vitamin standard that were run daily. Some concentrations were run in duplicate. Vitamin concentrations were then interpolated from the regression equation of the trend line of the corresponding standard curve. The theoretical method limits of detection for each vitamin, in each matrix, were calculated as the lowest calibrator injected into the HPLC, in ng, divided by the amount of sample injected, in mg (Supplemental Table 7).

How well-values in different tissue types compared to the liver standard was evaluated using linear regression and by examining the *R*^2^ and their *p*-value. Results with a *p*-value of 0.05 or less were considered statistically significant.

## Results

### Vitamin Concentrations in Liver, Heart, and Pectoral Muscle Samples

Minimum, maximum, mean, standard deviation, and median for retinol concentrations for hummingbird liver, heart, and pectoral muscle samples are reported in Table 1. Mean ± standard deviation (SD) concentration of retinol in liver samples (*n* = 20) was 269.0 ± 216.9 ppm. For the heart and pectoral muscle samples (*n* = 20), the mean ± SD concentrations were 1.8 ± 2.2 ppm and 0.3 ± 0.1 ppm, respectively.

**Table 1:** Summary statistics for retinol wet mass concentrations in liver, heart, and pectoral muscle samples from after-hatch year Anna's Hummingbird carcasses (*n* = 20).

**Tissue type**	**Minimum (ppm)**	**Maximum (ppm)**	**Mean (ppm)**	**Standard deviation (ppm)**	**Median (ppm)**
Male liver (*n* = 10)	69.8	794.4	303.3	208.7	308.5
Female liver (*n* = 10)	44.2	811.5	234.7	230.5	156.4
Total liver (*n* = 20)	44.2	811.5	269.0	216.9	202.6
Male heart (*n* = 10)	0.6	4.7	1.4	1.2	1.1
Female heart (*n* = 10)	0.2	9.6	2.1	2.9	1.2
Total heart (*n* = 20)	0.2	9.6	1.8	2.2	1.2
Male pectoral muscle (*n* = 10)	0.1	0.4	0.3	0.1	0.3
Female pectoral muscles (*n* = 10)	0.09	0.4	0.3	0.1	0.3
Total pectoral muscle (*n* = 20)	0.09	0.4	0.3	0.1	0.3

*For each tissue type, the minimum (ppm), maximum (ppm), mean (ppm), standard deviation (ppm), and median (ppm) were calculated for samples from male, female, and total hummingbirds (males and females)*.

Mean ± SD α-tocopherol concentrations in hummingbird samples (*n* = 20) were 6.9 ± 4.6 ppm, 5.5 ± 4.0 ppm, and 3.7 ± 2.2 ppm for liver, heart, and pectoral muscles, respectively. Minimum, maximum, mean, standard deviation, and median for α-tocopherol concentrations for liver, heart, and pectoral muscle samples are reported in Table 2. Only 19 birds were analyzed for α-tocopherol concentrations in the brain because one sample (Bird# 24) was lost during analysis. Mean ± SD α-tocopherol concentration in hummingbird brain samples (*n* = 19) was 9.1 ± 3.2 ppm.

**Table 2:** Summary statistics for α-tocopherol wet mass concentrations in liver, heart, pectoral muscle, and brain samples from after-hatch year Anna's Hummingbird carcasses (*n* = 20).

**Tissue type**	**Minimum (ppm)**	**Maximum (ppm)**	**Mean (ppm)**	**Standard deviation (ppm)**	**Median (ppm)**
Male liver (*n* = 10)	0.5	17.9	8.8	5.4	8.5
Female liver (*n* = 10)	0.4	9.7	5.0	2.9	5.1
Total liver (*n* = 20)	0.4	17.9	6.9	4.6	5.8
Male heart (*n* = 10)	0.4	14.2	7.4	4.4	7.3
Female heart (*n* = 10)	0.08	7.1	3.6	2.5	3.8
Total heart (*n* = 20)	0.08	14.2	5.5	4.0	4.8
Male pectoral muscle (*n* = 10)	2.3	9.6	4.9	2.1	4.4
Female pectoral muscle (*n* = 10)	0.1	4.8	2.5	1.6	2.3
Total pectoral muscle (*n* = 20)	0.1	9.6	3.7	2.2	3.7
Male brain (*n* = 9)	3.4	13.0	8.7	2.9	8.9
Female brain (*n* = 10)	4.8	15.9	9.5	3.6	8.5
Total brain (*n* = 19)	3.4	15.9	9.1	3.2	8.9

*One brain sample was not available for analysis, therefore n = 19 for brain samples. For each tissue type, the minimum (ppm), maximum (ppm), mean (ppm), standard deviation (ppm), and median (ppm) were calculated for samples from male, female, and total hummingbirds (males and females)*.

### Vitamin Concentration Relationships Among Liver, Heart, Pectoral Muscle, and Brain Samples

**Figure 1:**
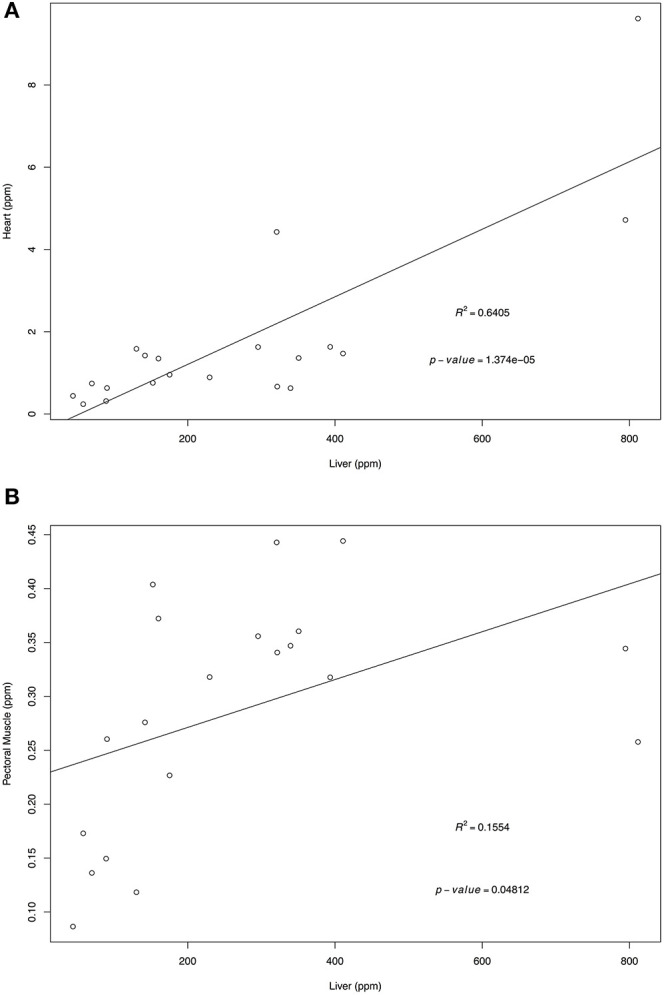
Regression of liver retinol wet mass concentrations (ppm) on heart **(A)** and pectoral muscle **(B)**. Tissues samples were obtained from after-hatch year Anna's Hummingbird carcasses (*n* = 20). Samples were analyzed by high performance liquid chromatography.

**Figure 2:**
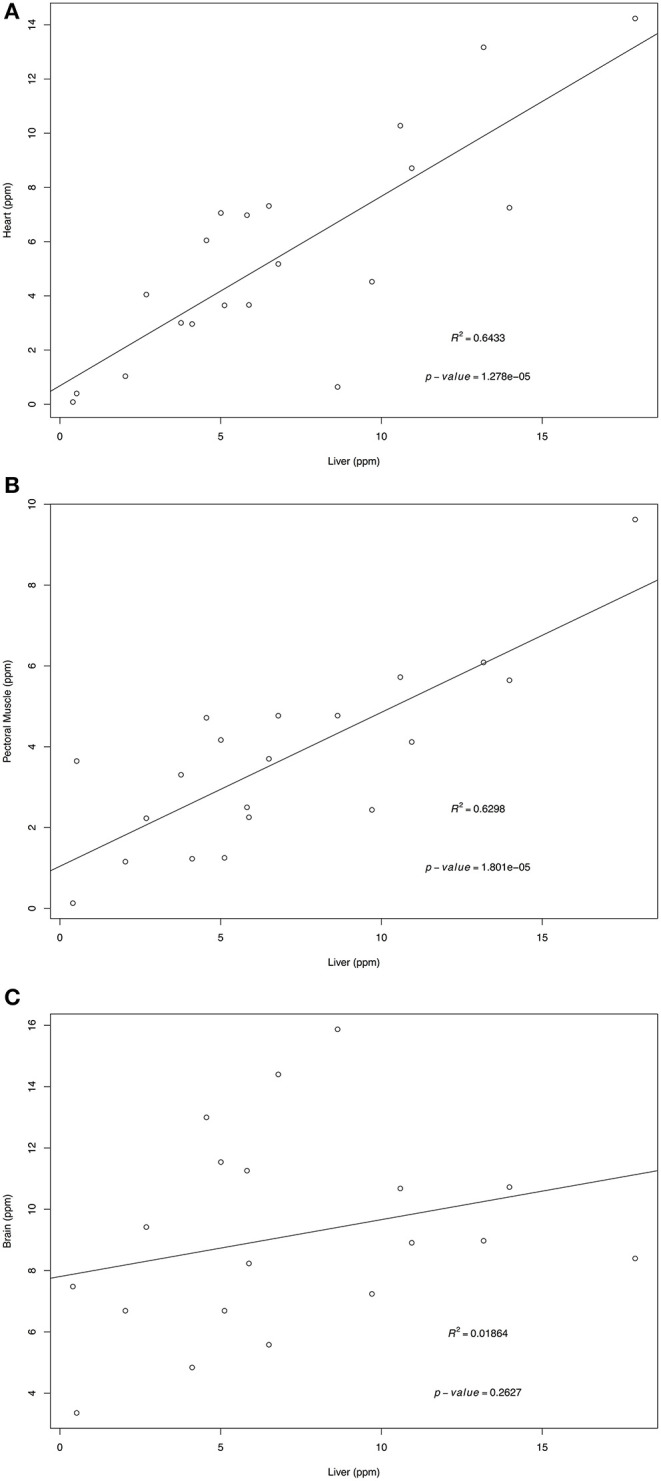
Regression of liver α-tocopherol wet mass concentrations (ppm) on heart **(A)**, pectoral muscle **(B)**, and brain **(C)**. Tissues samples were obtained from after-hatch year Anna's Hummingbird carcasses (*n* = 20). Samples were analyzed by high performance liquid chromatography.

Retinol and α-tocopherol concentrations of heart, pectoral muscle, and brain samples were regressed against the traditional tissue of choice, liver (Figures 1, 2). For retinol concentrations, the regression of values from heart samples on liver values had a statistically significant value *R*^2^ = 0.64 (*p* = 1.374e-05); the regression of pectoral muscle samples on liver was also significant (*p* = 0.048), but with a low *R*^2^ = 0.16. For α-tocopherol concentrations, the heart and liver regression also had a significant *R*^2^ = 0.64 (*p* = 1.278e-05) as did pectoral muscle (*R*^2^ = 0.63, *p* = 1.801e-05) whereas the regression of brain and liver samples was not significant (*R*^2^ = 0.02, *p* = 0.263).

### Validating HPLC at a Smaller Tissue Quantity

Means, standard deviations, and CVs were calculated for each chicken control batch and for the total overall batches (Supplemental Tables 3, 4). For all control samples (liver, heart, pectoral muscles, brain, solvent spike 1 ppm, and solvent spike 500 ppm), the CVs were 20% and under for both intra-assay and inter-assay evaluations.

The spiked quality control chicken tissue homogenates all had CVs of 20% or under. Results of the retinol and α-tocopherol concentrations recovered are listed in Supplemental Tables 5, 6.

## Discussion

This study developed an accurate analytical assay for quantifying retinol and α-tocopherol concentrations in small mass tissue samples, reported vitamin concentration ranges for hummingbird tissues with the intent that this information could help future rehabilitation efforts, and determined whether brain, heart, and pectoral muscle could accurately inform overall concentrations relative to the standard tissue liver. The brain was only considered for α-tocopherol analysis because hummingbirds commonly experience head trauma. Therefore, the authors were curious as to how α-tocopherol concentrations might be of benefit in the event of an injury. Retinol was not considered due to lack of resources and time.

The first phase of the study was to develop and validate an analytical method for measuring retinol and α-tocopherol in small mass tissue samples because most methods require large sample masses (8). Similar to another study that developed a method for quantifying trace elements in small mass tissue samples (10) the assay method for retinol and α-tocopherol has consistent performance. In addition, regardless of the variability in chicken brain tissue quality control sample masses (lowest mass of 0.05 g), α-tocopherol concentrations were very close to expected concentrations (CV = 6.7%), thus supporting the precision of the analytical method.

As expected, hummingbird liver samples contained the highest mean concentration of retinol. This finding was expected because retinol is a fat-soluble vitamin, which accumulates mostly in organ tissues (11). Heart and pectoral muscle samples followed in respect to their retinol concentrations. For α-tocopherol, brain samples had the highest mean α-tocopherol concentration. Hummingbirds have a remarkable metabolic rate, one of the highest mass-specific metabolic rates among homoeothermic vertebrates (12). As a result, they also have an increased generation of reactive oxidation species (ROS). Defense against lipid peroxidation and ROS involves many antioxidant components, but mainly α-tocopherol (13). The brain is a highly sensitive organ, and high concentrations of α-tocopherol help combat peroxidation. The original hypothesis was that pectoral muscles would generate more ROS than the brain and liver due to the energy demands needed to sustain a hovering flight pattern, and thus would have higher α-tocopherol concentrations. However, this was not the case as liver, heart, and pectoral muscle samples had the second, third, and fourth highest mean concentrations of α-tocopherol, suggesting the potential degree of lipid peroxidation and ROS generated by those tissues. The authors suspect that the liver is an organ with high metabolic demands, which may explain why the liver had higher α-tocopherol concentrations compared to other tissues. In addition, it was interesting that α-tocopherol concentrations were relatively high in brain tissue samples. The brain is also a metabolically active organ, but the higher α-tocopherol concentrations may have an additional benefit to hummingbirds that experience head trauma. Another theory is that the pectoral muscles have evolved for efficiency, therefore they do not generate as much ROS compared to other organs. Unfortunately, to the authors' knowledge, there are no published studies that have evaluated vitamin concentrations in brain, liver, and pectoral muscle samples from birds for which to compare. However, in sheep, liver was shown to have higher concentration of vitamin E, followed by heart and then skeletal muscle (14). Liver concentration was also increased in comparison to skeletal muscle in pigs (15).

For retinol, when comparing liver concentrations with other tissues, the significant regressions of heart and pectoral muscle samples on liver indicate that both tissues could be potential samples for determining retinol concentrations. Heart and pectoral muscles have the advantage of being more easily salvaged in a small carcass than the liver, and are less prone to tissue autolysis. Based on the *R*^2^-values, heart samples were better substitutes for liver samples compared to pectoral muscle samples.

For α-tocopherol, heart and pectoral muscle samples were also significant predictors of liver values, suggesting that either tissue can be utilized for assessing α-tocopherol status. However, the higher *R*^2^-value for heart samples suggest that they would be a better reflection of the liver concentrations than pectoral muscle samples. The high *p*-value and low *R*^2^-value for the brain samples indicate that the brain samples are not suitable for estimating liver α-tocopherol concentrations.

To evaluate species difference, the authors compared hummingbird retinol and α-tocopherol concentrations (mean ± SD) with chicken quality control sample concentrations (Supplemental Table 2) as quantified by the same analytical method. For retinol, the quality control chicken liver sample mean (482.2 ± 57.7 ppm) was higher than that for hummingbird samples (269.0 ± 216.9 ppm). However, the hummingbird pectoral muscle samples (0.3 ± 0.1 ppm) had the same mean concentration as the chicken quality control samples (0.3 ± 0.03 ppm). Surprisingly, hummingbird heart samples had a higher mean retinol concentration (1.8 ± 2.2 ppm) than the chicken quality control samples (0.2 ± 0.05 ppm), suggesting species differences in retinol storage.

For α-tocopherol, hummingbird pectoral muscle (3.7 ± 2.2 ppm) and brain (9.1 ± 3.2 ppm) samples had higher mean concentrations than chicken quality control samples (pectoral muscle: 1.2 ± 0.05 ppm, brain: 6.2 ± 0.4 ppm). This may be a reflection of a hummingbird's greater need for anti-oxidants compared to chickens (16) Chickens have a lower metabolic rate in comparison (12), so theoretically they would not need as robust of an anti-oxidant system to counteract generation of free-radicals. A surprising finding was that the mean α-tocopherol concentration for hummingbird heart samples (5.5 ± 4.0 ppm) was lower than the chicken samples mean (8.3 ± 1.4 ppm). A hummingbird's heart is a highly efficient and specialized organ, ensuring that oxygenated blood reaches the pectoral muscles to sustain a hovering flight pattern (17). It would be reasonable to expect that a higher anti-oxidant concentration (α-tocopherol) would be necessary to counteract the amount of ROS generated by the heart and the hummingbird's circulatory system, but this is not the case as seen in the data.

Establishing a retinol concentration range for quality control chicken liver samples was necessary as part of the assay validation, but also provided an opportunity to compare retinol chicken liver sample concentration from our study with published data. For our study, the mean retinol chicken liver concentration was 482.2 ± 57.7 ppm. This mean concentration is very different from previously reported values, which may be attributed to our small sample size. Across the literature, there is a large discrepancy in retinol concentrations. One manuscript reported the mean to be 370 ppm (converted from 370 RE mg/kg) (18) while another study found the mean to be 56.0 ± 38.9 ppm (converted from 5.60 ± 3.89 mg RE/100 g) (19). Published concentrations represent total retinol concentrations, including not only trans-retinol, but also retinyl palmitate, oleate, stearate, and linoleate as well. Our study employed a procedure that measured trans-retinol. However, a hydrolysis method was performed so that, in theory if all of the retinyl esters were hydrolyzed in the samples to retinol, the concentrations from our study would be equivalent to the RE seen in reported literature. In addition, the variation may be due to differences in study location, chicken age, amount of dietary retinol, breed of chicken, etc. For example, mean values for chicken livers of Britain was 97 ppm (9.7 mg RE/100 g), of the Slovak Republic was 170-200 ppm (17–20 mg RE/100 g), and of Germany was 335 ppm (33.5 mg RE/100 g) (19).

These same principles could be the source of variability in the hummingbird retinol concentrations as seen by the high standard deviation and the range of values (Table 1, Supplemental Table 8). Being a migratory species, the hummingbird carcasses probably represented different geographic regions, which would impact their diet. In addition, hummingbirds have age and sex differences in their diets which affect the ratio of nectar to insects consumed. Although the hummingbirds were all determined to be aged “AHY” (adult), we were unable to determine where in the reproductive cycle each bird was in, which can vastly impact the vitamin concentrations.

Vitamin E concentrations were measured as α-tocopherol in this study. Reported chicken liver mean α-tocopherol concentration values are scarce. One report found the mean concentration was 2.1 ppm (0.21 mg/100 g) (20). Again, the value is different from the mean α-tocopherol concentration obtained in this study, which was 9.3 ppm. The difference may be due to variation in diet and how much retinol was supplemented in the chickens' diets. Supplementing with retinol, which is commonly done in commercial feeds, has shown to have a linear depressive effect on α-tocopherol concentrations in the plasma and liver samples from both chicks and adult chickens (21, 22).

A limitation of this study was using free-ranging hummingbirds as animal subjects. This could cause a variation in the diet that the birds consumed and the environment that they inhabited. Dietary consumption can impact vitamin concentrations, especially vitamin E in the form of α-tocopherol. Geographic location and time of year can influence dietary consumption (23) and, since hummingbirds are migratory species, this presents even more of a challenge.

Prior to being presented to wildlife rehabilitation centers, all hummingbirds used in this study were free-ranging. These birds were opportunistically collected, which means they had an unknown health status at time of death. However, the data from this study at least serve as an initial reference point from which to build upon. The birds (*n* = 18) that were dead on presentation or were euthanized within 24 h would most likely better represent the nutrient status of free ranging birds. However, for 22 hummingbirds, the length in captivity could not be determined due to lack of management information. Therefore, these birds could have been fed a commercial diet while in captivity. Liver is a tissue matrix that has been documented to be especially sensitive to changes in dietary vitamin consumption (22). Sheep livers were seen to have a decrease in α-tocopherol liver within a week of being fed an α-tocopherol depleted diet (22). The same is seen for vitamin A when vitamin A was depleted for 3 weeks in turkeys (23). Ten hummingbirds used for retinol analysis and 12 hummingbirds used for α-tocopherol analysis were not documented as dead on arrival or euthanized within 24 h. It is possible that these birds, while in captivity, could have been fed a supplemental diet for an extended period of time, which would impact the results. In a study in which growing pigs experienced vitamin E supplementation and depletion, muscle was less likely to change with changes in vitamin dietary consumption, reflecting long-term nutritional history (24). Therefore, for the 22 cases with unknown histories, it may be more appropriate to use pectoral muscle to accurately reflect the vitamin concentration of free-ranging hummingbirds.

Future studies might benefit from evaluating tissues on a dry mass basis since hummingbird tissues desiccate rapidly following dissection. In addition, this method could possibly be used with plasma for quantifying vitamin concentrations antemortem, thus enabling determination of real time vitamin concentrations in live hummingbirds.

## Conclusion

A HPLC analytical method for measuring retinol and α-tocopherol concentrations in small mass tissue samples was established and validated. Baseline wet weight concentrations of retinol and α-tocopherol in hummingbird liver, heart, pectoral muscle, and brain samples were determined. Tissue analysis for any analyte is a challenge in hummingbirds given their small body size and the tendency for their tissues to rapidly autolyze post-mortem. Comparison among various tissue types was performed to determine which tissue best reflected liver sample concentrations so that other tissues could be considered in the case of liver autolysis. Heart samples best reflected liver concentrations for retinol, and both heart and pectoral muscle samples reflected liver concentrations for α-tocopherol. Having methods for quantifying retinol and α-tocopherol concentrations in hummingbird samples might help with conservation efforts and avoid hypervitaminosis and hypovitaminosis in the case of rehabilitated birds or hummingbirds being raised in captivity

## Data Availability Statement

All datasets presented in this study are included in the article/Supplementary Material.

## Ethics Statement

Ethical review and approval was not required for the animal study because permission for obtaining the hummingbird carcasses for scientific study was approved by the United States Fish and Wildlife Service (Permit: MB55944B-2) and the California Department of Fish and Wildlife (Permit: SC-013066).

## Author Contributions

LT, RP, LA, and GG contributed to conception and design of the study. SD and GG processed tissue samples and collected data. SD and LT wrote the original manuscript. JF wrote the R codes and guided data analysis. SD performed the statistical analysis. RB aided in specimen selection and collection. All authors contributed to manuscript revision and approved the submitted version.

## Conflict of Interest

The authors declare that the research was conducted in the absence of any commercial or financial relationships that could be construed as a potential conflict of interest.

## Footnotes

^1^CAHFS internal procedure, effective date 12/02/2018.

^2^Cornell Lab of Ornithology Birds of the World. Anna's Hummingbird (2020).
