# Supplementary material for: Concentrations of Retinol and α-Tocopherol in Tissue Samples From Anna's Hummingbirds (Calypte anna)
Source: Front Vet Sci. 2020 Sep 29;7:637. doi: 10.3389/fvets.2020.00637 (PMC7553074; doi:10.3389/fvets.2020.00637)
Supplement: Supplementary file 1 [file Data_Sheet_1.docx]

***Supplementary Material***

**Supplemental Table 1.** Summary of wet mass values for whole liver, heart, pectoral muscle, and brain samples for high performance liquid chromatography retinol and α-tocopherol analysis. Mean (g), range (g), and standard deviation (g) are provided for each tissue type.

| **Tissue Type** | **Mean Sample Mass (g)** | **Sample Ranges (g)** | **SD of Sample Masses (g)** |
| --- | --- | --- | --- |
| **Retinol Analysis** | | | |
| Liver (n=20) | 0.07 | 0.05-0.13 | 0.02 |
| Heart (n=20) | 0.09 | 0.05-0.13 | 0.02 |
| Pectoral Muscle (n=20) | 0.5 | 0.05-0.5 | 0.07 |
| **α-tocopherol Analysis** | | | |
| Liver (n=20) | 0.07 | 0.05-0.15 | 0.03 |
| Heart (n=20) | 0.10 | 0.05-0.13 | 0.02 |
| Pectoral Muscle (n=20) | 0.5 | 0.05-0.5 | 0.06 |
| Brain (n=19) | 0.09 | ***0.03**-0.13 | 0.03 |

*Mass for brain is highlighted to indicate the low mass of 0.03 g, which is lighter than the 0.05 g criteria for this study. The study was validated at 0.05 g. Recovery of α-tocopherol in tissue samples under 0.05 g is unknown. The concentrations for these low mass brain samples were included to have a complete data set. Caution should be used when interpreting this data.

**Supplemental Table 2.** Mean (ppm), standard deviation (ppm), range (mean + 2SD) for chicken tissue homogenates and solvent spiked samples used as quality control samples for retinol and α-tocopherol. Values are based upon wet mass.

| Vitamin | Tissue Type | Mean (ppm) | Standard Deviation (ppm) | Range (Mean + 2SD) |
| --- | --- | --- | --- | --- |
| Retinol | Chicken Liver  (n=15) | 482.2 | 57.7 | 366.9 – 597.4 |
|  | Chicken Heart  (n=15) | 0.2 | 0.05 | 0.1—0.03 |
|  | Chicken Pectoral Muscle  (n=14) | 0.3 | 0.03 | 0.2—0.3 |
|  | Solvent Spike 1 ppm  (n=5) | 1.08 | 0.08 | 0.9—1.2 |
|  | Solvent Spike 500 ppm  (n=5) | 513.3 | 53.4 | 406.6—620.1 |
| α-tocopherol | Chicken Liver  (n=11) | 9.3 | 0.6 | 8.1—10.6 |
|  | Chicken Heart  (n=11) | 8.3 | 1.4 | 5.6—11.1 |
|  | Chicken Pectoral Muscle  (n=11) | 1.2 | 0.05 | 1.1—1.3 |
|  | Chicken Brain  (n=9) | 6.2 | 0.4 | 5.4—7.1 |
|  | Solvent Spike 1 ppm  (n=5) | 1.0 | 0.08 | 0.8—1.2 |

**Supplemental Table 3.** Retinol mean (ppm), standard deviation (ppm), and intra-assay and inter-assay coefficients of variation (%) calculated from concentrations for quality control chicken liver, chicken heart, chicken pectoral muscle, solvent spike 1 ppm, and solvent spike 500 ppm samples. Values are based upon wet mass.

| Tissue Type | Batch Number | Number of Samples | Mean (ppm) | Standard deviation (ppm) | CV (%) |
| --- | --- | --- | --- | --- | --- |
| Chicken Liver | L1 | 5 | 518.9 | 45.8 | 8.8 |
|  | L2 | 5 | 512.6 | 25.7 | 5.0 |
|  | L3 | 7 | 416.2 | 48.6 | 11.7 |
|  | L4 | 10 | 413.1 | 20.5 | 5.0 |
|  | L5 | 2 | 524.0 | 23.7 | 4.5 |
|  | L6 | 2 | 407.3 | 18.6 | 4.6 |
|  | L7 | 2 | 414.0 | 17.9 | 4.3 |
|  | L8 | 2 | 430.9 | 23.3 | 5.4 |
|  | L9 | 2 | 427.9 | 33.7 | 7.9 |
|  | Total | 37 | 448.9 | 56.7 | 12.6 |
| Chicken Heart | H1 | 5 | 0.2 | 0.01 | 7.8 |
|  | H2 | 5 | 0.2 | 0.02 | 8.7 |
|  | H3 | 2 | 0.2 | 0.02 | 8.5 |
|  | H4 | 2 | 0.2 | 0.05 | 20.4 |
|  | H5 | 2 | 0.2 | 0.009 | 4.8 |
|  | H6 | 2 | 0.2 | 0.005 | 2.2 |
|  | Total | 18 | 0.2 | 0.03 | 14.2 |
| Chicken Pectoral | PM1 | 10 | 0.3 | 0.01 | 5.1 |
| Muscle | PM2 | 2 | 0.3 | 0.008 | 2.4 |
|  | PM3 | 4 | 0.3 | 0.006 | 2.0 |
|  | PM4 | 2 | 0.3 | 0.008 | 2.8 |
|  | Total | 18 | 0.3 | 0.03 | 10.4 |

| Solvent Spike 1 ppm | BS1.1 | 5 | 1.1 | 0.08 | 7.0 |
| --- | --- | --- | --- | --- | --- |
|  | BS1.2 | 2 | 1.3 | 0.3 | 19.1 |
|  | BS1.3 | 1 | N/A | N/A | N/A |
|  | BS1.4 | 1 | N/A | N/A | N/A |
|  | BS1.5 | 1 | N/A | N/A | N/A |
|  | BS1.6 | 1 | N/A | N/A | N/A |
|  | BS1.7 | 1 | N/A | N/A | N/A |
|  | BS1.8 | 1 | N/A | N/A | N/A |
|  | BS1.9 | 1 | N/A | N/A | N/A |
|  | BS1.10 | 1 | N/A | N/A | N/A |
|  | BS1.11 | 1 | N/A | N/A | N/A |
|  | BS1.12 | 1 | N/A | N/A | N/A |
|  | Total | 17 | 1.1 | 0.1 | 11.5 |
| Solvent Spike 500 ppm | BS 500.1 | 5 | 513.3 | 53.4 | 10.4 |
|  | BS 500.2 | 1 | N/A | N/A | N/A |
|  | BS 500.3 | 1 | N/A | N/A | N/A |
|  | BS 500.4 | 1 | N/A | N/A | N/A |
|  | BS 500.5 | 1 | N/A | N/A | N/A |
|  | BS 500.6 | 1 | N/A | N/A | N/A |
|  | BS 500.7 | 1 | N/A | N/A | N/A |
|  | Total | 11 | 486.9 | 46.0 | 9.4 |

**Supplemental Table 4.** α-tocopherol mean (ppm), standard deviation (ppm), and intra-assay and inter-assay coefficients of variation (%) calculated from concentrations for quality control chicken liver, chicken heart, chicken pectoral muscle, chicken brain, and solvent spike 1 ppm samples. Values are based upon wet mass.

| Tissue Type | Batch Number | Number of Samples | Mean (ppm) | Standard deviation (ppm) | CV (%) |
| --- | --- | --- | --- | --- | --- |
| Chicken Liver | L1 | 2 | 9.2 | 0.3 | 3.3 |
|  | L2 | 1 | N/A | N/A | N/A |
|  | L3 | 1 | N/A | N/A | N/A |
|  | L4 | 5 | 9.2 | 0.6 | 6.5 |
|  | L5 | 2 | 9.9 | 1.0 | 9.9 |
|  | L6 | 2 | 10.2 | 0.1 | 1.0 |
|  | L7 | 2 | 9.5 | 0.1 | 1.3 |
|  | L8 | 2 | 10.4 | 0.005 | 0.05 |
|  | L9 | 2 | 10.0 | 0.7 | 7.1 |
|  | L10 | 2 | 9.0 | 0.6 | 6.2 |
|  | Total | 21 | 9.6 | 0.7 | 6.9 |
| Chicken Heart | H1 | 3 | 8.1 | 0.9 | 11.3 |
|  | H2 | 3 | 8.9 | 0.8 | 8.9 |
|  | H3 | 2 | 8.1 | 0.3 | 4.3 |
|  | H4 | 3 | 7.2 | 1.3 | 17.6 |
|  | H5 | 2 | 9.2 | 0.007 | 0.08 |
|  | H6 | 2 | 7.8 | 0.05 | 0.6 |
|  | Total | 15 | 8.4 | 1.2 | 14.4 |
| Chicken Pectoral Muscle | PM1 | 3 | 1.2 | 0.03 | 2.7 |
|  | PM2 | 3 | 1.3 | 0.07 | 5.5 |
|  | PM3 | 2 | 1.2 | 0.005 | 0.4 |
|  | PM4 | 3 | 1.2 | 0.04 | 3.6 |
|  | PM5 | 2 | 1.2 | 0.02 | 1.7 |
|  | PM6 | 1 | N/A | N/A | N/A |
|  | PM7 | 2 | 1.3 | 0.003 | 0.2 |
|  | Total | 16 | 1.2 | 0.07 | 5.4 |
| Chicken Brain | B1 | 7 | 6.2 | 0.4 | 5.7 |
|  | B2 | 2 | 6.5 | 0.7 | 11.2 |
|  | B3 | 2 | 6.3 | 0.09 | 1.4 |
|  | B4 | 2 | 6.7 | 0.6 | 8.2 |
|  | Total | 13 | 6.3 | 0.4 | 6.7 |
| Solvent Spike 1 ppm | BS1.1 | 5 | 1.0 | 0.08 | 7.6 |
|  | BS1.2 | 1 | N/A | N/A | N/A |
|  | BS1.3 | 1 | N/A | N/A | N/A |
|  | BS1.4 | 1 | N/A | N/A | N/A |
|  | BS1.5 | 1 | N/A | N/A | N/A |
|  | BS1.6 | 1 | N/A | N/A | N/A |
|  | BS1.7 | 1 | N/A | N/A | N/A |
|  | BS1.8 | 1 | N/A | N/A | N/A |
|  | BS1.9 | 1 | N/A | N/A | N/A |
|  | BS 1.10 | 1 | N/A | N/A | N/A |
|  | BS1.11 | 1 | N/A | N/A | N/A |
|  | BS1.12 | 1 | N/A | N/A | N/A |
|  | Total | 16 | 1.1 | 0.1 | 12.4 |

**Supplemental Table 5.** Results for retinol spiked chicken tissue homogenates (liver, heart, pectoral muscle, and brain tissue). Liver samples were spiked with 500 ppm of retinol; heart samples were spiked with 1ppm of retinol; and pectoral muscle was spiked with 1 ppm of retinol. Ppm, adjusted ppm, mean of adjusted ppm, standard deviation of adjusted ppm, and coefficient of variation of the adjusted ppm (%) are reported. Results are based on wet mass.

| Tissue Type | Sample Number | ppm | Adjusted ppm | Recovery (%) | Mean Adjusted (ppm) | Standard deviation of Adjusted (ppm) | CV of Adjusted (%) |
| --- | --- | --- | --- | --- | --- | --- | --- |
| Chicken Liver | 1 | 570.8 | 301.4 | 60 |  |  |  |
|  | 2 | 606.7 | 337.3 | 67 |  |  |  |
|  | 3 | 657.9 | 388.5 | 78 |  |  |  |
|  | 4 | 716.7 | 447.3 | 89 |  |  |  |
|  | 5 | 649.3 | 379.9 | 76 |  |  |  |
|  | 6 | 640.1 | 370.7 | 74 |  |  |  |
|  | 7 | 683.0 | 413.6 | 83 |  |  |  |
|  | 8 | 619.7 | 350.3 | 70 |  |  |  |
|  | Total |  |  |  | 373.6 | 45.3 | 12.1 |
| Chicken Heart | 1 | 0.9 | 0.5 | 54 |  |  |  |
|  | 2 | 0.8 | 0.5 | 48 |  |  |  |
|  | 3 | 0.9 | 0.6 | 59 |  |  |  |
|  | 4 | 0.9 | 0.6 | 56 |  |  |  |
|  | 5 | 0.8 | 0.5 | 50 |  |  |  |
|  | 6 | 0.9 | 0.5 | 52 |  |  |  |
|  | 7 | 0.8 | 0.4 | 44 |  |  |  |
|  | 8 | 0.8 | 0.5 | 50 |  |  |  |
|  | Total |  |  |  | 0.5 | 0.05 | 9.4 |
| Chicken Pectoral Muscle | 1 | 1.1 | 0.7 | 75 |  |  |  |
|  | 2 | 0.8 | 0.5 | 50 |  |  |  |
|  | 3 | 1.1 | 0.8 | 75 |  |  |  |
|  | 4 | 1.1 | 0.8 | 77 |  |  |  |
|  | 5 | 1.1 | 0.8 | 76 |  |  |  |
|  | 6 | 0.9 | 0.6 | 56 |  |  |  |
|  | 7 | 0.8 | 0.5 | 51 |  |  |  |
|  | 8 | 1.1 | 0.8 | 75 |  |  |  |
|  | Total |  |  |  | 0.7 | 0.1 | 18.5 |

**Supplemental Table 6.** Results for α-tocopherol spiked chicken tissue homogenates (liver, heart, pectoral muscle, and brain tissue). Liver samples were spiked with 10 ppm of α-tocopherol; heart samples were spiked with 10 ppm of α-tocopherol**;** and brain samples were spiked with 10 ppm of α-tocopherol. Ppm, adjusted ppm, mean of the adjusted ppm, standard deviation of the adjusted ppm, and coefficient of variation of the adjusted ppm (%) are reported. Results are based on wet mass.

| Tissue Type | Sample Number | ppm | Adjusted ppm | Recovery (%) | Mean Adjusted (ppm) | Standard deviation (ppm) | CV (%) | |
| --- | --- | --- | --- | --- | --- | --- | --- | --- |
| Chicken Liver | 1 | 21.0 | 8.4 | 84 |  |  | |  |
|  | 2 | 21.4 | 8.9 | 89 |  |  | |  |
|  | 3 | 17.9 | 5.3 | 53 |  |  | |  |
|  | 4 | 21.6 | 9.1 | 91 |  |  | |  |
|  | 5 | 19.7 | 7.1 | 71 |  |  | |  |
|  | 6 | 20.6 | 8.0 | 80 |  |  | |  |
|  | 7 | 20.4 | 7.9 | 78 |  |  | |  |
|  | 8 | 17.8 | 5.2 | 52 |  |  | |  |
|  | Total |  |  |  | 7.5 | 1.5 | | 20 |
| Chicken Heart | 1 | 12.5 | 5.3 | 53 |  |  | |  |
|  | 2 | 12.4 | 5.2 | 52 |  |  | |  |
|  | 3 | 12.4 | 5.3 | 53 |  |  | |  |
|  | 4 | 10.3 | 3.2 | 32 |  |  | |  |
|  | 5 | 12.4 | 5.2 | 52 |  |  | |  |
|  | 6 | 11.7 | 4.6 | 46 |  |  | |  |
|  | 7 | 11.1 | 4.0 | 40 |  |  | |  |
|  | 8 | 11.3 | 4.1 | 41 |  |  | |  |
|  | Total |  |  |  | 4.6 | 0.8 | | 17.2 |
| Chicken Pectoral | 1 | 2.2 | 0.9 | 89 |  |  | |  |
|  | 2 | 2.2 | 0.9 | 92 |  |  | |  |

| Muscle | 3 | 2.1 | 0.9 | 86 |  |  |  |
| --- | --- | --- | --- | --- | --- | --- | --- |

|  | 4 | 2.0 | 0.8 | 78 |  |  |  |
| --- | --- | --- | --- | --- | --- | --- | --- |
|  | 5 | 2.2 | 1.0 | 96 |  |  |  |
|  | 6 | 2.2 | 0.9 | 89 |  |  |  |
|  | 7 | 2.1 | 0.9 | 87 |  |  |  |
|  | 8 | 2.1 | 0.8 | 82 |  |  |  |
|  | Total |  |  |  | 0.9 | 0.05 | 6.6 |
| Chicken Brain | 1 | 16.0 | 9.5 | 95 |  |  |  |
|  | 2 | 16.0 | 9.5 | 95 |  |  |  |
|  | 3 | 15.7 | 9.2 | 92 |  |  |  |
|  | 4 | 16.1 | 9.6 | 96 |  |  |  |
|  | 5 | 17.5 | 10.9 | 109 |  |  |  |
|  | 6 | 16.2 | 9.7 | 97 |  |  |  |
|  | Total |  |  |  | 9.7 | 0.6 | 6.3 |

**Supplemental Table 7.** Theoretical method limits of detection for each vitamin and tissue type in ppm (wet mass).

| **Tissue Type** | **Theoretical Method Limit of Detection (ppm)** |
| --- | --- |

| **Retinol** |
| --- |

| Liver | 0.8 |
| --- | --- |
| Heart | 0.09 |
| Pectoral Muscle | 0.5 |

| **α-tocopherol** |
| --- |

| Liver | 0.03 |
| --- | --- |
| Heart | 0.04 |
| Pectoral Muscle | 0.05 |
| Brain | 0.1 |

**Supplemental Table 8.** Retinol concentrations (ppm) for hummingbird liver, heart, and pectoral muscle samples. These values are based on wet mass. Bird #, sex, values are listed for each sample.

| **Bird #** | **Sex** | **Liver (ppm)** | **Heart (ppm)** | **Pectoral Muscle (ppm)** |
| --- | --- | --- | --- | --- |
| 1 | Male | 410.9 | 1.5 | 0.4 |
| 2^†^ | Male | 229.8 | 0.9 | 0.3 |
| 3^†^ | Male | 339.6 | 0.6 | 0.3 |
| 4^†^ | Male | 69.8 | 0.7 | 0.1 |
| 5 | Male | 350.7 | 1.4 | 0.4 |
| 6^†^ | Male | 90.4 | **0.6*** | 0.3 |
| 7^†^ | Male | 130.3 | **1.6*** | 0.1 |
| 8^†^ | Male | 295.5 | **1.6*** | 0.4 |
| 9^†^ | Male | 794.4 | **4.7*** | 0.3 |
| 10^†^ | Male | **321.5*** | **0.7*** | 0.3 |
| 11 | Female | 320.8 | 4.4 | 0.4 |
| 12 | Female | 160.2 | 1.3 | 0.4 |
| 13 | Female | 393.5 | 1.6 | 0.3 |
| 14 | Female | 811.5 | 9.6 | 0.3 |
| 15 | Female | 152.6 | 0.8 | 0.4 |
| 16^†^ | Female | 175.4 | 1.0 | 0.2 |
| 17 | Female | 89.1 | 0.3 | 0.1 |
| 18^†^ | Female | 58.1 | 0.2 | 0.2 |
| 19 | Female | 142.0 | 1.4 | 0.3 |
| 20 | Female | 44.2 | 0.4 | 0.1 |

*Concentrations for heart samples from birds #6-10, and the concentration for liver sample from bird #10 are bolded to indicate that their 1 ppm retinol solvent spiked samples exceeded the expected concentrations. However, the chicken quality control samples that were run in conjunction with these samples were within established ranges. Heart and liver sample values for these birds were accepted, but caution should be used when interpreting data.

^†^These birds were either presented dead on arrival or were euthanized within 24 hours of being presented to the wildlife center. The management of the other birds was not reported.

**Supplemental Table 9.** α-tocopherol concentrations (ppm) for hummingbird liver, heart, pectoral muscle, and brain samples. These values are based upon wet mass. Bird #, sex, and values are listed for each sample.

| **Bird #** | **Sex** | **Liver (ppm)** | **Heart (ppm)** | **Pectoral Muscle (ppm)** | **Brain (ppm)** |
| --- | --- | --- | --- | --- | --- |
| 21 | Male | 0.5 | 0.4 | 3.6 | 3.4 |
| 22^†^ | Male | 17.9 | 14.2 | 9.6 | 8.4 |
| 23^†^ | Male | 13.2 | 13.2 | 6.1 | 9.0 |
| 24 | Male | 3.8 | 3.0 | 3.3 | No Value |
| 25 | Male | 6.5 | 7.3 | 3.7 | 5.6 |
| 26^†^ | Male | 10.6 | 10.3 | 5.7 | 10.7 |
| 27^†^ | Male | 10.9 | 8.7 | 4.1 | 8.9 |
| 28^†^ | Male | 14.0 | 7.3 | 5.6 | 10.7 |
| 29 | Male | 5.9 | 3.7 | 2.3 | 8.2 |
| 30 | Male | 4.6 | 6.0 | 4.7 | 13.0 |
| 31^†^ | Female | 5.8 | 7.0 | 2.5 | 11.3 |
| 32 | Female | 6.8 | 5.2 | 4.8 | 14.4 |
| 33 | Female | 4.1 | 3.0 | 1.2 | 4.8 |
| 34 | Female | 9.7 | 4.5 | 2.4 | 7.2 |
| 35^†^ | Female | 5.1 | 3.6 | 1.2 | 6.7 |
| 36^†^ | Female | 5.0 | 7.1 | 4.2 | 11.5 |
| 37 | Female | 8.6 | 0.6 | 4.8 | **15.9*** |
| 38 | Female | 2.0 | 1.0 | 1.2 | 6.7 |
| 39 | Female | 0.4 | 0.08 | 0.1 | **7.5*** |
| 40 | Female | 2.7 | 4.0 | 2.2 | 9.4 |

The brain sample from bird #24 was lost during the homogenization phase. This study employed the use of the wet mass. Since the tissue masses were so small, desiccation in the freezer may have impacted the results and may attribute to the slight fluctuations in the results. *Birds #37 and #39 had brain samples that weighed below 0.05g, the minimum mass requirement in this study, and were noted to be especially dry at the time of analysis but were above 0.05 g. All solvent spikes of brain samples had over 120% recovery. However, the chicken brain control samples were well within the range. The chromatographic graph suggested that there was an interference that may have produced the increased recovery, which led to the out-of-range values. The values were accepted, but caution should be used when interpreting this data.

^†^These birds were either presented dead on arrival or were euthanized within 24 hours of being presented to the wildlife center. The management of the other birds was not reported.
